# Therapeutic Targeting of Autoreactive B Cells: Why, How, and When?

**DOI:** 10.3390/biomedicines9010083

**Published:** 2021-01-16

**Authors:** Zachary C. Stensland, John C. Cambier, Mia J. Smith

**Affiliations:** 1 Barbara Davis Center for Diabetes, Department of Pediatrics, University of Colorado School of Medicine, Aurora, CO 80045, USA; zachary.stensland@cuanschutz.edu; 2 Department of Immunology and Microbiology, University of Colorado School of Medicine, Aurora, CO 80045, USA; john.cambier@cuanschutz.edu

**Keywords:** B cells, autoimmunity, self-antigen, antigen-specific therapies, tolerance, B lymphocytes, anergy

## Abstract

B lymphocytes play critical roles in the development of autoimmunity, acting as autoantibody manufacturers, antigen-presenting cells, and producers of cytokines. Pan-B cell depletion has demonstrated efficacy in treatment of many autoimmune disorders, but carries with it an unfavorable safety profile due to global immune suppression. Hence, attention has turned to the potential of autoantigen-specific B cell targeted therapies, which would deplete or silence pathogenic self-antigen-reactive cells while sparing B cells needed for immune defense. Here, we discuss the antigen-specific B cell-targeted approaches that are under development or are under consideration, that could be employed to allow for more precise therapy in the treatment of autoimmunity. Lastly, we discuss some of the challenges associated with antigen-specific B cell targeting that may impact their clinical applicability.

## 1. Introduction: The “Why?”

B lymphocytes play a crucial role in development of autoimmunity. In many cases autoantibodies produced by the differentiated daughters of autoreactive B cells mediate disease, such as in systemic lupus erythematosus (SLE), Graves disease, and myasthenia gravis. In other cases autoantibodies, but not B cell function, are dispensable for disease, such as in type 1 diabetes (T1D) and multiple sclerosis (MS). B cells likely participate in disease through non-mutually exclusive ways, including antigen-presentation to T cells, pro-inflammatory cytokine release, and antibody production. Hence, it is not surprising that over the past few decades B cell directed therapies, such as rituximab (anti-CD20), have demonstrated clinical efficacy in treatment of a number of autoimmune diseases [1,2,3,4,5,6].

Nonetheless, pan-B cell depletion therapies, such as rituximab and belimumab (anti-BAFF), are not optimal. These therapeutics are non-specific, targeting both pathogenic and non-pathogenic B cells, resulting in global suppression of humoral immunity. It appears that while pre-existing immunity is spared, development of immunity to newly encountered pathogens, such as SARS-CoV-2, would not. In addition, while B cell depletion typically lasts 6–12 months following cessation of treatment, in some instances individuals never fully recover their B cell compartment [7,8,9]. Lastly, anti-CD20 therapies target the majority of B cell subsets but spare antibody producing plasma cells, which do not express CD20 on their cell surface. This fact could explain the varying response to treatment seen in diseases such as SLE and rheumatoid arthritis (RA) in which reduction of autoantibodies is typically incomplete [10,11]. Hence, attention by pharmaceutical companies and funding agencies has turned to development of antigen-specific therapies that target the pathogenic B cells, ideally including plasma cells, while leaving the remainder of the immune system functioning normally. However, the development of such novel therapeutics has been hampered by the inherent difficulties in detecting and studying rare antigen-specific B cells that occur at frequencies of <1/100 in the normal B cell repertoire. Here we discuss approaches under development to target autoreactive B cells and the challenges associated with their use in the clinic.

## 2. Methods to Target Antigen-Specific B Cells: The “How?”

**Figure 1:**
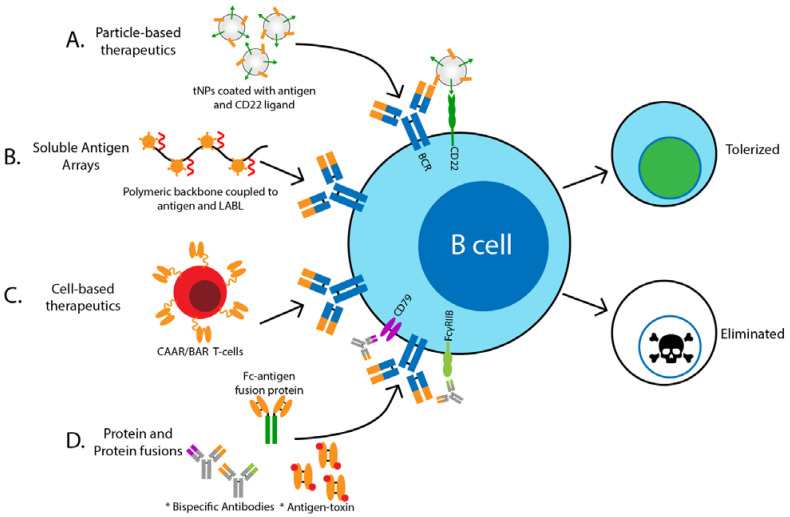
Overview of antigen-specific B cell targeting therapeutics under development. Various methods have been developed to target antigen-specific B cells for either tolerance induction or removal from the repertoire. These methods include (**A**) particle-based therapies, (**B**) soluble antigen arrays, (**C**) cell-based therapies, and (**D**) protein and protein fusions. These methods combine delivery and binding of antigen and associated toxic or inhibitory cargo to the target cell by virtue of recognition by the antigen receptor (BCR). Abbreviations: tolerogenic nanoparticles (tNPs), cell adhesion inhibitor peptide (LABL), chimeric auto-antibody receptor (CAAR), B-cell targeting antigen receptor (BAR)* indicates methodologies that are currently unpublished, but warrant consideration.

The basic principle behind antigen-specific targeting of B cells include delivery and binding of antigen and associated toxic cargo to the target cell by virtue of recognition by the antigen receptor (BCR) (Figure 1). This requires coupling of the antigen to some form of toxic inhibitory, or apoptosis-inducing molecule or protein, so that when the B cell of interest binds its cognate antigen complex, it is rendered unresponsive or killed. Many different strategies may be employed to accomplish this goal.

### 2.1. Particle-Based Therapeutics

Generation of tolerogenic nanoparticles (tNPs) to treat autoimmunity has recently gained popularity. Synthetic polymer-based nanoparticles have proven safe and offer flexibility in determining the appropriate structure and surface properties, while also allowing efficient carriage of drug and proteins to target cells. In addition, studies have demonstrated tNPs are especially affective at targeting antigen-presenting cells (APCs), such as dendritic cells and macrophages, due to the inherent ability of these APCs to selectively endocytose. For the majority of studies using tNPs to treat autoimmune disease, nanoparticles containing known self-peptides are administered to mice and engulfed by APCs that then present them in the context of MHC to autoreactive T cells in the absence of co-stimulation, resulting in T cell tolerance [12,13,14,15,16]. However, in order to directly target autoreactive B cells, native autoantigenic epitopes recognized by these cells would need to be displayed on the surface of the nanoparticle. In addition, incorporation of some inhibitory molecule or toxic drug may be required. Multivalent antigen complexes can be very effective at cross-linking and activating B cells compared to soluble antigen [17,18], which would result in unwanted activation of self-reactive B cells. However, if properly designed, such complexes can induce B cell unresponsiveness that could be exploited therapeutically.

Macauley et al. utilized liposomal nanoparticles that display both antigen and glycan ligands of CD22, an inhibitory co-receptor on the surface of B cells that when ligated and co-localized with the BCR (Figure 1A), induces recruitment of phosphatases that inhibit BCR signaling [19,20]. Using a mouse model for hemophilia, in which production of anti-Factor VIII (FVIII) antibodies prevents the effective treatment of mice with exogenous FVIII, this group found that these nanoparticles coupled to FVIII, selectively targeted B cells for apoptosis. Deletion of FVIII-reactive B cells resulted in decreased production of anti-FVIII antibodies that allowed successful administration of FVIII to hemophilia mice to prevent bleeding [20]. Similar results were found by another group who used tNPs containing FVIII and rapamycin, an inhibitor of the mTOR pathway and potent immune modulator [12]. While studies using this type of technology have been limited to models of hemophilia, it seems reasonable it could be extended to other models of autoimmunity in the future.

### 2.2. Soluble Antigen Arrays

Another method to deliver self-antigen for induction of tolerance has involved soluble multivalent antigen arrays, in which a polymeric backbone is coupled with autoantigen and cell adhesion inhibitory (LABL) peptides (Figure 1B) [21,22]. Using this method, Hartwell et al. have shown that co-delivery of the self-antigen PLP with LABL was efficacious in preventing development of experimental autoimmune encephalomyelitis (EAE), a murine model of MS. They demonstrated that these multivalent soluble antigen arrays target antigen-specific B cells directly through binding to the B cell receptor (BCR), rendering the B cell anergic (unresponsive) [21,23]. Using a similar technology to couple insulin to the polymeric background, the same group demonstrated specific binding to insulin-reactive B cells and resulting desensitization [24]. Though it appears promising, it remains to be seen if targeting insulin-reactive B cells using this method results in protection from disease development. Future studies should be aimed at determining the efficacy of multivalent soluble antigen arrays at treating or preventing other forms of autoimmunity.

### 2.3. Cell-Based Therapeutics

With the advent of cell-based therapeutics, including generation of chimeric antigen receptor (CAR)-T cells to treat cancer, some researchers have begun to utilize this technology to treat autoimmunity. Generally speaking, CAR-T cells express a chimeric receptor composed of an extracellular domain consisting of a monoclonal antibody fragment that recognizes a specific antigen important in disease pathogenesis. The cytoplasmic portion of the CAR contains signaling domains, most commonly derived from CD28 and CD3ξ, that mediate T cell activation upon antigen recognition. This results in targeted killing or suppression of the cell, depending on whether it is an NK, Teff or Treg cell. In the NOD mouse model of autoimmune diabetes, it is known that presentation of the insulin B chain peptide residues 9–23 in the context of MHCII (I-A^g7^) by APCs to autoreactive T cells is important in development of disease [25]. Zhang et al. generated a monoclonal antibody (mab287) that recognizes the insulin I-A^g7^-B:9–23 complex and showed that it effectively blocked recognition by B:9–23 reactive T cells and delayed development of diabetes in NOD mice [26]. More recently, she generated a 287-CAR CD8+ T cell that targets APCs presenting I-A^g7^-B:9-23 and found that it is relatively specific and effective at killing APCs presenting I-A^g7^-B:9-23 and delays development of disease [27]. Since antigen presentation by islet-reactive B cells is essential for diabetes development in the NOD, it seems likely that 287-CAR-T cell act by deletion of insulin-reactive B cells [28,29]. Future studies should be carried out to determine if this is indeed the case.

Other studies have demonstrated specific targeting of autoreactive B cells using CAR-T cell technology (Figure 1C). Recently, “BAR” (B-cell-targeting Antibody Receptor) Tregs were shown to prevent anti-FVIII antibody production in the mouse model of hemophilia [30,31]. In these studies Treg cells bearing chimeric receptors comprised of the immunodominant FVIII epitope linked with the CD28-CD3ξ signaling domains were shown to suppress the anti-FVIII antibody response in vivo in mice by induction of tolerance in FVIII-reactive B cells, and allowed therapeutic administration of FVIII to stop the bleeding [30]. The authors suggested that this effect was mediated by direct FVIII-specific BAR-Treg suppression of FVIII-reactive B cells and induction of tolerance in T cells important in B cell activation. Similarly, Parvathaneni and Scott generated FVIII-specific BAR-CD8+ T cells, which specifically targeted FVIII-reactive B cells for deletion and reduced anti-FVIII antibody formation in hemophilic mice [32]. These studies demonstrate generation of BAR-Treg and BAR-CD8+ T cells are effective at targeting harmful antibody producing B cells and may prove to be useful in treatment of other autoimmune diseases.

Similarly, Ellebrecht et al. demonstrated chimeric autoantibody receptor (CAAR) T cells could be used to treat pemphigus vulgaris (PV) by selectively depleting desmoglein 3 (Dsg3)-reactive B cells, which are known to mediate disease via autoantibody production [33]. This group transduced human T cells with lentiviral vectors to express a chimeric receptor with an extracellular Dsg3 and intracellular signaling CD137-CD3ξ domains. When administered to a mouse model of PV, the CAAR-T cells depleted circulating Dsg3-secreting B cells, reduced anti-Dsg3 IgG levels in serum, and prevented mucosal blistering [33]. More recently, this same group completed a preclinical study of Dsg3-CAART for the treatment of mucosal PV in humans. Their study found Dsg3-CAAR T cells specifically killed human anti-Dsg3 B cells from PV patients ex vivo and showed activity consistent with a threshold dose in vivo [34]. Results from this study has supported an investigational new drug application for a Phase I clinical trial. If approved, results from this study will be an important step toward determining therapeutic potential of BAR/CAAR T cells to treat human autoimmunity.

### 2.4. Protein and Protein Fusions

Although recent studies have predominately used particle-based therapeutics, such as nanoparticles or cells to deliver antigen to B cells, soluble protein therapeutics are on the horizon. Benefits of these biologics include their small size, bioavailability, solubility, and increased specificity for targeted B cells. In a proof of concept study, Henry et al. used an anti-insulin monoclonal antibody (mab123) to target insulin-reactive B cells whose BCR is occupied by endogenous insulin using a VH125 heavy chain transgenic NOD mouse model of diabetes [35]. Insulin-reactive B cells were significantly reduced after injection of mab123 in vivo, likely through FcγR mediated depletion. Moreover, since mab123 does not recognize insulin bound to the insulin receptor [36], mice remained normoglycemic in nondiabetic mice. Importantly, this group demonstrated that systemic administration of mab123 to WT NOD mice resulted in protection from diabetes development [35].

While this method demonstrates the feasibility of using monoclonal antibodies to target antigen-specific B cells, it requires the BCR of autoreactive B cells to be occupied by antigen. In addition, it would not target B cells that recognize an insulin epitope bound by the 123 antibody, nor immediately adjacent epitopes due to steric hindrance. Moreover, there is concern that administration could result in formation of insulin antibody complexes and ultimately impair the action of physiologic action insulin. Hence to overcome these challenges, Akston Biosciences has created a designer Fc-insulin fusion protein, AKS-107, in which a hormonally inactive insulin is fused to an IgG Fc to specifically target insulin-reactive B cells for complement and FcγR mediated killing or induction of anergy (Figure 1D). This approach has the added advantage that the IgG Fc should extend the in vivo half-life of the drug by interaction with FcRN. Promising pre-clinical studies in mice demonstrate AKS-107 induces specific silencing of insulin-reactive B cells and can prevent autoimmune diabetes (unpublished), providing the basis for a first-in-human Phase 1 clinical trial of AKS-107. Results will greatly inform our understanding of the safety and potential applications of autoantigen-IgG Fc fusions to treat T1D, with implications for other autoimmune diseases in which pathogenic antigen-specific B cells are implicated.

While not targeting antigen specific B cells, antibody-based therapies that induce B cell unresponsiveness (anergy) through engagement of FcγRIIb have shown efficacy in treatment of autoimmunity and avoid the harmful side effects of total B cell depletion. FcγRIIb is an inhibitory surface receptor expressed primarily by B cells that when co-engaged with the BCR mediates recruitment of phosphatases that suppress B cell signaling [37]. Xencore Inc developed XmAb5871, which is a humanized mAb that binds to CD19 in the BCR complex and is engineered to have increased affinity for FcγRIIb. Preclinical studies have shown that co-engagement of CD19 and FcγRIIb by XmAb5871 suppresses B cell activation, inhibits upregulation of CD86, and suppresses antibody production [38,39]. Recently a Phase 2 clinical trial in SLE patients demonstrated that XmAb5871 is well tolerated and significantly suppressed disease recurrence [40]. Another antibody that exploits the inhibitory function of FcγRIIb is the bispecific antibody, PRV-3279 (formally known as MGD010), which binds to CD79b, a signaling component of the BCR, and FcγRIIb [41]. The first-in-human study of MGD010 demonstrated it is tolerated, does not cause B cell depletion but induces downregulation of surface BCR and suppression of BCR-mediated Ca^2+^ mobilization, indicative of B cell anergy [42]. Plans to address its potential use in autoimmunity are currently in development. Still, another approach emulates the ability of chronic low avidity antigen exposure, as occurs with soluble autoantigens in vivo, to induce B cell anergy. Antibodies lacking antibody-dependent cell-mediated cytotoxicity (ADCC) and complement fixing ability that are reactive with CD79 induce polyclonal anergy. As shown by the Cambier group and others, these antibodies are effective in mouse models of lupus, T1D, RA and MS (EAE) [43,44] (Cambier unpublished). While the above candidate therapeutics silence all B cells, the technology could be exploited to generate antibodies that engage the BCR in an antigen-specific manner, similar to AKS-107, which would result in tolerance induction of pathogenic B cells while sparing non-pathogenic B cells (Figure 1D).

Another carrier-free antigen-specific therapeutic on the horizon incorporates a drug or toxin directly conjugated to a self-antigen, similar to antibody-drug conjugates (ADCs) currently in use to treat some forms of cancer [45,46,47,48]. In principle, antigen-toxin conjugates would bind specifically to the BCR on pathogenic self-reactive B cells, inducing death by virtue of concentration and cellular uptake of the toxin (Figure 1D). In a proof-of-principle study, Pickens et al. demonstrated that conjugation of the self-antigen peptide, PLP_139-152_, to the corticosteroid, dexamethasone, led to protection from development of EAE in mice with increased efficacy compared to dexamethasone treatment alone [49]. While this study utilized a self-antigen fragment and not full-length protein and did not analyze the effects on the immune system in depth, it demonstrates the potential of this type of therapeutic to prevent or delay autoimmunity. Preliminary studies in the Cambier lab have shown that insulin conjugated to the toxin, mitoxantrone, induces specific deletion of murine insulin-binding B cells in vitro (unpublished). Future studies are needed to fully address the therapeutic potential of antigen-toxin conjugates in the treatment of autoimmune disease.

## 3. Challenges to Antigen-Specific B Cell Therapy: The “When?”

Despite their infancy, therapies that target antigen-specific B cells for the treatment of autoimmunity (Table 1) show promise and carry an enhanced safety profile relative to current therapies that induce total B cell depletion. However, there are important considerations and potential limitations that should be acknowledged that may limit their clinical efficacy. For example, in autoimmune disorders, such as SLE, many patients develop autoantibodies against more than one antigen [50] and, therefore, simultaneous targeting of B cells with various specificities may be needed for maximal efficacy. On the other hand, while rituximab trials have demonstrated a clear benefit of B cell depletion in MS, knowledge of which autoantigens are important in disease pathogenesis are still not defined [51,52]. In addition, studies in T1D, MS, RA, and SLE have suggested epitope spreading can occur, such that autoimmune disease could be initiated by one antigen but propagated during chronic autoimmunity by a different antigen, thus making it especially difficult to treat someone with established disease [53,54,55,56,57,58].

Importantly, proper timing of therapy may be crucial. In T1D, RA, and SLE, patients can develop autoantibodies many years before onset of disease and, therefore, targeting of antigen-specific B cells once disease is confirmed may be too late. Conversely, many healthy individuals generate autoantibodies but never have autoimmune disease, and therefore, preemptive treatment based on autoantibody positivity may be unnecessary for some individuals. Other aspects to consider include the potential need for multimodal therapies that target pathogenic B cells as well as T cells and/or cytokines for the most effective treatment. Moreover, while cell-based antigen-specific therapies, such as BAR and CAAR T cells, have proven to be beneficial, the time and cost of ex vivo manipulation of autologous patient cells may limit their translational potential to treat human autoimmunity.

Ultimately, the largest theoretical impediment to the success of therapies that specifically target autoreactive B cells is the fact that by the time the patient reaches the clinic, and thus the first opportunity for therapeutic intervention, they are producing high levels of autoantibody. These autoantibodies can be expected to bind and neutralize the candidate autoantigen therapeutics before they reach their B cell target. Utility of autoreactive B cell-specific therapies may be relegated to situations in which genetic risk dictates a certainty that disease will develop, yet autoantibodies are not yet being produced.

## 4. Conclusions

Despite many concerns and challenges, therapeutic silencing of autoreactive B cells remains a very attractive prospect. Thus, it is an exciting time for the development of antigen-specific therapeutics to treat autoimmunity. Current and future studies will inform our understanding of the therapeutic efficacy of these treatments to prevent, manage, and/or cure autoimmune disease.

## Figures and Tables

**Table 1:** Summary of antigen-specific B cell therapies to date.

Disease Model	Method	Results	References
Hemophilia	tNPs coated with FVIII and CD22 ligands	FVIII-specific B cell deletion	[20]
	tNP with FVIII encapsulated with rapamycin	FVIII-specific B cell deletion	[12]
	FVIII-BAR Treg	FVIII-specific B cell tolerance and antibody suppression	[27]
	FVIII-BAR CD8+ T cell	FVIII-specific B cell deletion	[30]
EAE	Soluble antigen array with PLP + LABL	PLP-reactive B cell anergy	[21,23]
Pemphigus Vulgaris	Dsg3-CAAR T cell	Dsg3-specific B cell deletion	[33,34]
Autoimmune diabetes	Soluble antigen array with insulin + LABL	Insulin-specific B cell anergy ex vivo	[24]
	287-CAR CD8+ T cell	I-Ag7-B:9–23 presenting APC deletion	[27]
	mab123	Insulin-binding B cell deletion	[35]
	Fc-insulin fusion protein (AKS-107)	Insulin-binding B cell deletion/anergy	unpublished
	Insulin-toxin	Insulin-binding B cell deletion	unpublished

## Data Availability

Not applicable

## Abbreviations

SLE	systemic lupus erythematosus
T1D	type 1 diabetes
MS	multiple sclerosis
RA	rheumatoid arthritis
BCR	B cell receptor
tNPs	tolerogenic nanoparticles
APC	antigen-presenting cell
MHC	major histocompatibility complex
EAE	experimental autoimmune encephalomyelitis
FVIII	Factor VIII
CAR	chimeric antigen receptor
ITAM	immunoreceptor tyrosine-based activation motif
PV	pemphigus vulgaris
Dsg3	desmoglein 3
NOD	non-obese diabetic
BAR	B-cell-targeting Antibody Receptor
CAAR	chimeric auto-antibody receptor
Treg	T regulatory cell
Teff	T effector cell
NK	natural killer cell
ADCC	antibody-dependent cell-mediated cytotoxicity
ADC	antibody-drug conjugate
